# An integrated proteomics analysis of bone tissues in response to mechanical stimulation

**DOI:** 10.1186/1752-0509-5-S3-S7

**Published:** 2011-12-23

**Authors:** Jiliang Li, Fan Zhang, Jake Y Chen

**Affiliations:** 1 Department of Biology, Purdue School of Science, Indiana University Purdue University Indianapolis (IUPUI), Indianapolis, IN 46202, USA; 2 Indiana University School of Informatics, Indianapolis, IN 46202, USA; 3 Indiana Center for Systems Biology and Personalized Medicine, Indianapolis, IN 46202, USA; 4 Department of Computer and Information Science, Purdue University School of Science, Indianapolis, IN 46202, USA

**Keywords:** Bone Stress, Biomarker Discovery, Pathway Analysis, Tandem Mass Spectrometry

## Abstract

Bone cells can sense physical forces and convert mechanical stimulation conditions into biochemical signals that lead to expression of mechanically sensitive genes and proteins. However, it is still poorly understood how genes and proteins in bone cells are orchestrated to respond to mechanical stimulations. In this research, we applied integrated proteomics, statistical, and network biology techniques to study proteome-level changes to bone tissue cells in response to two different conditions, *normal loading *and *fatigue loading*. We harvested ulna midshafts and isolated proteins from the control, loaded, and fatigue loaded Rats. Using a label-free liquid chromatography tandem mass spectrometry (LC-MS/MS) experimental proteomics technique, we derived a comprehensive list of 1,058 proteins that are differentially expressed among normal loading, fatigue loading, and controls. By carefully developing protein selection filters and statistical models, we were able to identify 42 proteins representing 21 Rat genes that were significantly associated with bone cells' response to quantitative changes between normal loading and fatigue loading conditions. We further applied network biology techniques by building a fatigue loading activated protein-protein interaction subnetwork involving 9 of the human-homolog counterpart of the 21 rat genes in a large connected network component. Our study shows that the combination of decreased anti-apoptotic factor, Raf1, and increased pro-apoptotic factor, PDCD8, results in significant increase in the number of apoptotic osteocytes following fatigue loading. We believe controlling osteoblast differentiation/proliferation and osteocyte apoptosis could be promising directions for developing future therapeutic solutions for related bone diseases.

## Introduction

Bone tissues are sensitive to its mechanical environment [1]. It is well accepted that the presence of a reasonable level of mechanical stress on bones (known as *normal loading*) could enhance bone formation and maintain a healthy bone mass [2]. Prolonged absence of normal loading on bones--usually associated with extended physical inactivity due to injuries--could decrease bone formation and increase bone resorption, eventually leading to bone loss and disuse osteoporosis. When the level of mechanical stimulations exceeds the normal amount for an extended period of time, a stress condition known as *fatigue loading *could occur. In fatigue loading, *microdamage *such as small cracks in bone tissues may appear, triggering a cascade of bone remodeling processes that attempt to repair damaged bone tissues via sequential bone resorption and formation [3]. When fatigue loading conditions are not recognized early and addressed, the risks for bone injuries and bone diseases will increase. Therefore, understanding the constituents and functions of molecular repertoires involved in fatigue loading has been a central focus of study in molecular biology of the bone.

It still remains unknown what all the mechanically-sensitive genes and proteins in bone cells under mechanical stress are and how their differential expressions are regulated [4]. Past research identified osteoblast as being recruited to bone surfaces to form new bones in response to loading [5]. In fatigue loading conditions, the migration of osteoblast to the bone surface is known to co-occur with migrations of osteoblast progenitors and osteoblast to bone damaged areas, thus activating bone remodeling process and damage repairs [6-11]. This process requires temporal coordination of osteoblast and osteoblast to repair damaged bone tissues. Therefore, osteoblast-associated genes were reported and presumed to be involved with different levels of mechanical stimulation signals [12]. Several biochemical studies have also suggested that anabolic mechanical stimulation may increase the expression of *c-fos*, *osteopontin*, *COX-2*, *guanosine triphosphatases *(*GTPases*), *adenylate cyclase*, *phospholipase C *(*PLC*), and *mitogen-activated protein kinases *(*MAPKs*), which can further lead to elevated expression of bone anabolic factors such as *prostaglandins *and *Nitric oxide *(See reference [13] for a review).

In this work, we performed the first proteomic study of mechanical loading of bone tissues using Rat as an animal model. Prior to our study, large-scale functional genomics analysis of the activation of bone remodeling process were performed in a few microarray studies [14,15]. While these earlier studies suggested osteocyte apoptosis and Wnt signaling pathways were two critical biological processes involved, proper controls against normal loading conditions were not performed in those experimental studies. It was not clear what mRNA level changes observed in fatigue loading were shared in common with normal loading. Nor is it clear whether the biological processes observed at the mRNA expression level could overlook critical protein changes, since many recent studies revealed that large-scale gene expression and proteomics tend to complement (instead of significantly overlap) with each other [16,17]. Elucidating proteomics level changes, particularly when integrated with prior findings of genes and new models developed at the molecular signaling network/pathway level, can lead to new insights on bone mechanical stress and development of novel molecular biomarkers.

## Experimental procedures

### Design of bone loading experiments using rat models

**Figure 1:**
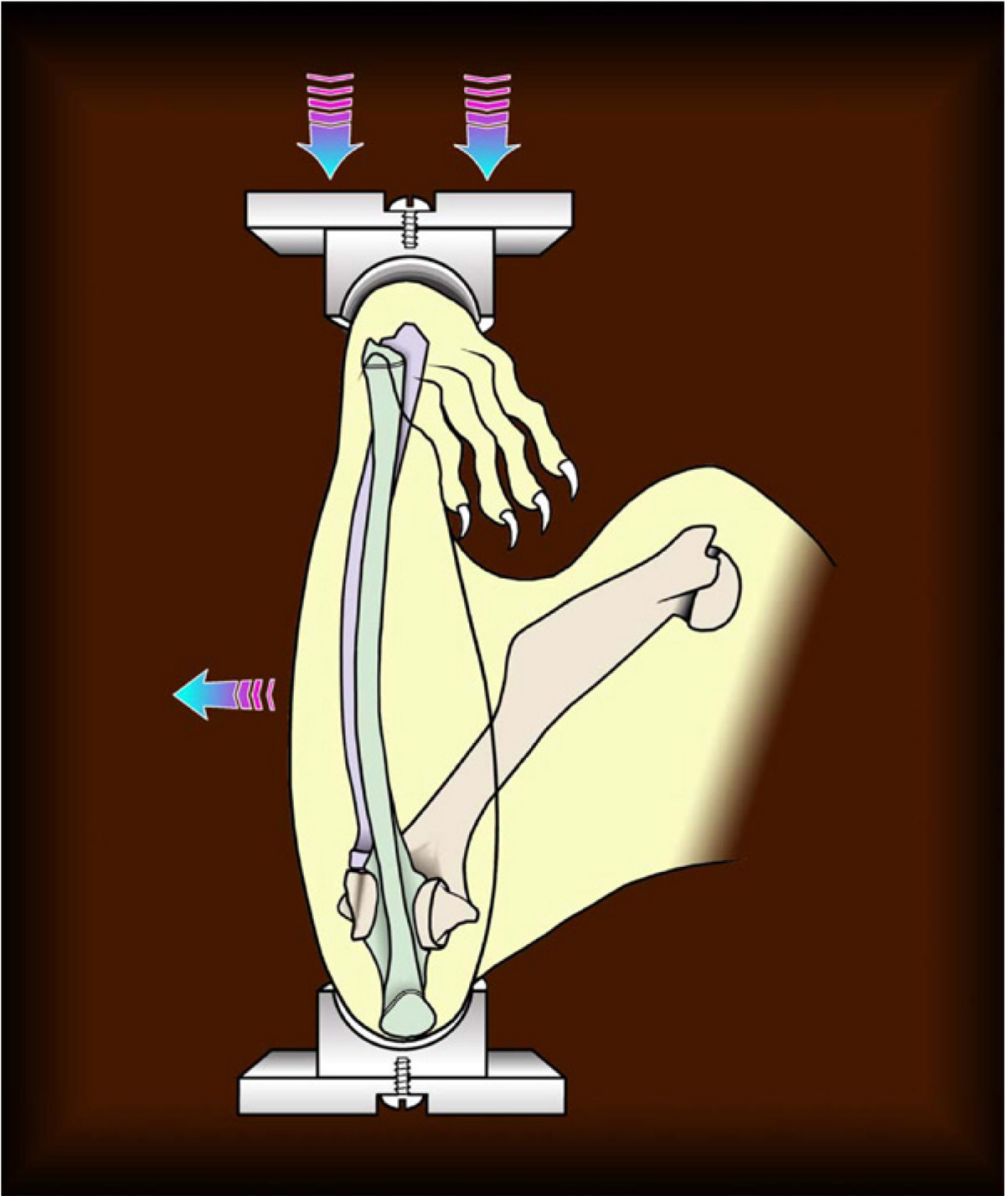
**An illustration of the ulnar axial compression loading system to study the effects of different levels of mechanical stress on bones in animal models**.

In order to study proteomics profile differences in living bone tissues, an ulnar axial compression loading system was chosen (see illustration in Figure 1). The system allows loading experimentation at different stress levels for animal models [6,10,11].

Female Sprague-Dawley Rat (age: 6 months; weight: 250-300 grams) were purchased from Harlan (Indianapolis, Indiana, USA). Animals were acclimatized for two weeks and housed in environmentally controlled rooms in Laboratory Animal Resource Center (LARC) of Indiana University School of Medicine and fed standard Rat chow and water *ad libitum*. All the procedures performed in this study were in accordance with the Indiana University Animal Care and Use Committee Guideline.

Nine animals were divided randomly into 3 groups: control (CTRL), loading (L) and fatigue loading (FL) groups. All the animals were anesthetized with an intraperitoneal injection of ketamine (60 mg/kg; Ketaset^®^--Fort Dodge Animal Health, Fort Dodge, IA) and xylazine (7.5 mg/kg; Sedazine^®^--Fort Dodge Animal Health, Fort Dodge, IA). The animals in the control group were sacrificed 96 hours post-injection without being subject to mechanical loading. The right ulnae of the remained animals were loaded or overloaded based on treatment groups. The animals in the loading group were loaded with a peak force of 20 N for 360 cycles and then sacrificed at 96 hours after the loading session. For the animals in fatigue loading group, one bout of loading with a peak force of 20 N at 2 Hz was not stopped until 10-15% stiffness loss. The overloaded animals were also sacrificed at 96 hours after the loading session.

Load was applied using a load-controlled, electromagnetic loading device. Total loading cycles was adjusted through the connected load controller. Stiffness loss during the loading procedure was observed through continuous monitoring of displacement of the arm on the loading device using a CCD Laser Displacement Sensor (LK Series, Keyence Corp. Osaka, Japan).

### Liquid chromatography coupled tandem mass spectrometry proteomics analysis

The ulnae were dissected out immediately and cleaned of all muscle and connective tissue after all the Rats were sacrificed. Both of 5-mm proximal and distal ends of the ulnae were removed. The remaining ulna midshafts were snap frozen in liquid nitrogen and stored at -80°C until protein isolation. For total protein isolation, Rat ulna midshafts were shattered and ground to a fine powder under liquid nitrogen using mortars and pestles. There were three groups (The control, loading and fatigue loading groups), three samples per group, and two HPLC injections per sample (Table 1).

**Table 1:** The experimental design for proteomics analysis of bone loading in rat

	Samples	Replicates	*Injection runs (Subtotals)*
CTRL	3	2	*6*
L	3	2	*6*
FL	*3*	*2*	*6*

The LC-MS/MS experiment consists of 3 groups × 3 samples × 2 replicates = 18 LC/MS injections run in random order. The three groups are: Controls (CTRL), Loaded (L), and Fully-Loaded (FL).

Label-free protein identification and protein quantitative analysis services were performed by professionals at the Protein Analysis and Research Center/Proteomics Core of Indiana University School of Medicine, co-located at Monarch Life Sciences, Inc, Indianapolis. For a thorough review of the principle and method developed at Monarch, refer to the review by Wang *et al *[18].

The protein identification tasks were analyzed using standard commercial-strength protocols and commercial software packages developed at Monarch, which have supported many scientific research case studies in areas including proteomics studies, biomarker discovery, and bioinformatics analysis, e.g., [19-21]. Briefly, Tryptic peptides were analyzed using Thermo-Finnigan linear ios-trap mass spectrometer (LTQ) coupled with a HPLC system. Peptides were eluted with a gradient from 5 to 45% Acetonitrile developed over 120 minutes and data were collected in the *triple-play *mode (MS Scan, zoom scan, and MS/MS scan). The acquired raw peak list data were generated by XCalibur (version 2.0) using default parameters and further analyzed by an algorithm using default parameters described by Higgs *et al *[22]. MS database searches were performed against the combined protein data set from International Protein Index (IPI; version 1.2) [23] and the non-redundant NCBI-nr human protein database (2005 version), which totaled 22,180 protein records. The resulting MS/MS data were searched using SEQUEST Cluster from Thermo Scientific (bundled with BioWorks software suite version 2.70 based on the original SEQUEST algorithm [24]). During search, we set the number of missed cleavages permitted to be 2. We search fixed modifications to be Iodoethanol on Cys and variable modifications to be Oxidation on Met. The mass tolerance for precursor ions were set at 2 Da and the mass tolerance for fragment ions were set at 0.7 Da. For novel protein that could not be positively identified by SEQUEST, we used the de novo sequencing function of the BioWorks software to obtain peptide sequence information for the collision-induced dissociation (CID) spectra. Carious data processing filters for protein identification were applied to keep only peptides with the XCorr score above 1.5 for singly charged peptides, 2.5 for doubly charged peptides, and 3.5 for triply charged peptides. These XCorr scores were set according to linear discriminant analysis similar to that described in DTASelect (version 2.0) to control false-positive rate at below 5% levels. These empirical thresholds were validated in large data sets processed by Monarch in similar conditions and peptide identification parameters. The false positive rates of these large-scale studies under the used parameters were estimated from the number and quality of spectral matches to the decoy database.

Protein quantification tasks were also conducted using software developed at Monarch Life Sciences, Inc. First, all extracted ion chromatograms (XICs) were aligned by retention time. Each aligned peak were matched by precursor ion, charge state, fragment ions from MS/MS data, and retention time within a one-minute window. Then, after alignment, the area-under-the-curve (AUC) for each individually aligned peak from each sample was measured, normalized, and compared for relative abundance--all as described in [22]. The normalization methods by Higgs *et al *[22] were used, and the data were then transformed back to the original scale. Here, a linear mixed model generalized from individual ANOVA (Analysis of Variance) was used to quantify protein intensities and calculate statistical significance. In principle, the linear mixed model considers three types of effects when deriving protein intensities based on weighted average of quantile-normalized peptide intensities: 1) *group effect*, which refers to the fixed non-random effects caused by the experimental conditions or treatments that are being compared; 2) *sample effect*, which refers to the random effects (including those arising from sample preparations) from individual biological samples within a group; 3) *replicate effect*, which refers to the random effects from replicate injections from the same sample preparation. Standard statistical data pre-processing techniques, including quantile normalization and randomization of measurement orders, were applied first to eliminate technical bias due to random variations from biological samples and their replicates. The model fitting was performed in the SAS software (version 9) using PROC MIXED. The REML method was used as a fit mechanism and degrees of freedom were computed using the Satterthwaite method. The RANDOM statement was used to model the covariance with the NOBOUND parameter option in the PROC statement. The p-value estimates the proportion of times a change at least as big as evaluated will be observed if in fact there is no real change. All the p-values were then transformed into q-values that estimate the False Discovery Rate (FDR) [25].

### Homologous gene mapping of rat and human proteins

Due to the lack of protein-protein interaction data coverage in Rat, we map all Rat protein-encoding genes to their human gene homolog to take advantage of large sets of protein interaction data available in human. The homologous gene mapping involved four steps. First, we extracted all the Rat protein identifiers (IPI number and protein GI accessions) from the sequence annotation field of the proteomics search results. Second, we downloaded Rat IPI reference database version 1.2, which contains 38,873 sequence identifier mapping relationships among Rat Swissprot IDs, sequence accession numbers, and gene names. Third, we downloaded NCBI Homologene release 49.1. We filtered out genes from other organisms to include proteins only from Rat and human. After applying the filter, 14,558 remained in the homologene groups, which contain homology mapping relationships between 15,125 Rat genes and 14,753 human genes. We defined a "homolog gene match" between a Rat gene and a human gene as each pair found within the same homologene group. In the fourth step, we map the matched human genes back to human proteins, using Uniprot sequence annotation files. Note that the mapping between Rat protein to human protein based on gene homology relationships has the limitation of aggregating all alternative spliced protein isoforms together. However, this will not be a major concern, since the majority protein-protein interaction data are collected based on gene-level experimentation data and therefore do not offer isoform-level resolution anyway.

### Method for selecting candidate significantly differentially-expressed proteins

For candidate proteins, we refer to the list of proteins that satisfies statistical protein-selecting filters but still needs further scrutiny before a subset of them can be confirmed as biologically relevant. It is tempting to control false positives using high *FC *threshold and *q-value *(false discovery Rate adjusted *p-value*) when we try to select candidate proteins that are differentially expressed with statistically rigor. For example, the following threshold filter (the *F1 *filter) was suggested by the proteomics analysis software by default to control possible false positives that may arise due to potential sources of variability (estimated to be up to 15%) from different sample and experimental errors:

$$F1:\text{FC}\left(x|i\right)\geq \text{1}.\text{5}\&q-\text{value}\left(x|i\right)<0.0\text{5}$$

While a stringent filter is generally necessary for proteomics experiments, protein expression level changes in proteomics experiments are generally expected to be smaller than those often observed in expression microarrays, because changes in signaling proteins or regulatory proteins are expected to be subtle in general. In addition, the problem with applying default filters directly is that these filters fail to take into account of data that may be highly correlated from controlled comparative experiments with more than two conditions. In our case, we have three conditions FL for fatigue loading, L for normal loading, and CTRL for normal controls. If we can observe high degree of correlation of results that occur in FL vs. CTRL and in F vs. CTRL, the FC requirement and q-value requirement may be both relaxed to allow more interesting proteins that change barely in the "twilight zone" of >10%, as long as these proteins can be further validated using additional computational or experimental techniques.

Therefore, in complementary to fold change filter in F1, we developed a second experimental filter (the *F2 *filter) to select candidate proteins that changed significantly above 10% (FC ≥ 1.1) to show up, when we try to compare two *similar *conditions, FL_vs_L (Fatigue Loading against Normal Loading), in which data for L_vs_CTRL (Fatigue Loading against Controls) and FL_vs_CTRL (Normal Loading against Controls) are also available:

*F2*: FC (*x*|FL_vs_L) ≥ 1.1 *and*

*q*-value(*x*|FL_vs_CTRL)*q-value(*x*|L_vs_CTRL) < 0.0025 *and*

*p*-value(*x*|FL_vs_CTRL) < 0.05 *& p*-value(*x*|L_vs_CTRL) < 0.05

Here in this F2 filter, in addition to relaxing the FC threshold, we also modified how we should apply statistical *q-value*. Here, we introduce a concept that we'll refer to as the *triangulation property *of comparable analysis. Briefly, this property is met if and only if pairwise comparison results from three conditions, for example, CTRL, L, and FL, are consistent among themselves. In other words, we say a triangulation property exists among CTRL-L-FL if and only if proteins passing FL_vs_CTRL and L_vs_CTRL q-value filters with FC changes of *f1 *and *f2 *respectively are the same set of proteins that pass FL_vs_L with and same q-value filter and a FC threshold of *f1/f2 *independently. In fact, no proteomics search software that we know today guarantee such triangulation property due to inherent errors in the model that estimates statistical significance of peptides and proteins. In fact, we understand that the q-value was derived from a more stringent statistical model in early years of proteomics licensed from Eli Lilly (private communication with Dr. Mu Wang, who provided the proteomics service for this experiment). Therefore, we developed an easy-to-understand meta-analysis method, *q-value triangulation method*, in the F2 filter, so that we can rely primarily on better-understood p-value statistics. In this method, we assume the p-value calculations of two independent experiments, FL_vs_CTRL and L_vs_CTRL, are generally reliable and therefore can be controlled at 0.05. The *q-value *triangulation calculation for FL_vs_L is done by multiplying the respective *q-values *for FL_vs_CTRL and L_vs_CTRL comparisons controlled at the 0.05^2 = 0.0025 level. The reason why the p-values are chosen comparing to the control samples rather than comparing FL vs L is that comparing to the control samples with our statistic method can reduce baseline noise in proteomics data and detect weak patterns.

### Normality probability plot calculation

To determine normality of the residual distribution, we use the normal probability plot to calculate the *normal quantiles *of all values in *Residue (i), or Res_FL_L*. The values and the normal quantiles are then plotted against each other. Normal quantiles are computed using the f-value, *f_i _*, which is calculated as:

$$f_{i}=\frac{i-0.5}{n}$$

where *i *is the index of the value and *n *is the number of values. The *normal quantile*, *q(f)*, for a given f-value is the value for which *P[X <= q] = f *, where *X *is a standard normally distributed variable [26].

### Creation of bone tissue stimulated protein sub-networks

Differentially expressed candidate Rat proteins, which we successfully mapped to human proteins through homologous gene matching, are used as *seed proteins *to build a protein-protein interaction subnetwork. We derive this protein interaction sub-network using a nearest-neighbor expansion method initially described in [27]. In summary, we searched the seed proteins against a human protein-protein interaction database. We include additional proteins in this subnetwork if and only if these additional proteins are found to directly interact with at least one seed protein. The protein-protein interactions involved are also collected into the subnetwork. If the subnetwork does not form a large connected graph, the biological functional distance among such seed proteins would be regarded as high. On the other hand, if the subnetwork does form a large connected graph, the biological functional distance among these seed proteins would be very close. The sub-network offers a good model to integrate proteomics results, from which drug target may be developed [20,27]. Since the seed proteins used are all proteins that are quantitatively changed under the FL_vs_L condition, this subnetwork is essentially an activated protein signaling network specific to bone cells' response to mechanical stress.

We use the Human Annotated and Predicted Protein Interaction (HAPPI) database [28] (http://bio.informatics.iupui.edu/HAPPI/) to retrieve high-quality protein interacting. We choose a human protein interaction database due to limited protein-protein interaction data available for Rat and the fact that Rat and human share the majority of biological processes in common. The HAPPI database is an open-access web-based relational database that contains a comprehensive collection of computer-annotated human protein-protein interactions involving 10,592 human proteins (identified by UniProt ID). Data in the HAPPI database are derived from both experimental data sources and computational predictions publicly available. Different from most protein-protein interaction databases, reliability of protein-protein interaction information is provided in the HAPPI database as *H score*s, which range between 0 to 1 or a quality star rank grade of 1, 2, 3, 4 and 5. Increased protein interaction grade from 1 to 5 have been shown to be associated with improved quality of physical interacting proteins and decreased amount of non-physical interactions found primarily in text mining or gene co-expression studies [29]. For this study, we only use protein interactions in the HAPPI database with star grade of 3 and higher (consisting of more than 280,000 human protein interactions of primarily physical interactions), which are comparable to the overall quality of HPRD, a much smaller reference human protein interaction database commonly used in bioinformatics.

#### Visualization of differentially expressed protein sub-network

To perform interaction network visualization, we used an internally developed software platform, ProteoLens [30], which can be freely downloaded from http://bio.informatics.iupui.edu/proteolens/. ProteoLens is a biological network data mining and annotation platform that supports both standard GML files and relational data in Oracle or PostgreSQL Database Management System. It is a scalable data-driven biological network visualization software that enables expert bioinformatics users to browse database schemas and tables, filter and join relational data using SQL queries, and customize data fields to be visualized as network graphs.

## Results

### Cellular changes in bone tissues after mechanical stimulations

**Figure 2:**
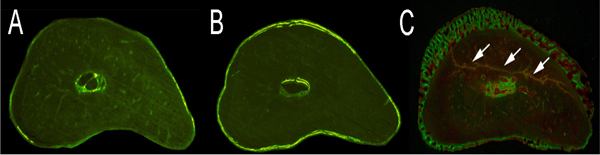
**Cellular changes of bone tissues under control, normal loading, and fatigue loading conditions**. A: Control condition (no loading); B: Normal loading condition. The thick staining at the perimeter of bone tissues indicates bone formation; C: Fatigue loading condition. The microdamage (indicated by arrows) and bone formation at the peripherals of bone tissues are clearly visible.

In Figure 2, we show a comparison of histological changes for bone tissues under control, normal loading, and fatigue loading conditions. In Figure 2A, we show a control without any mechanical stimulations. In Figure 2B, we show that bone formation in female SD Rat is significantly increased compared with the control, when one bout of axial loading of the ulna with a peak force of 20 N at 2 Hz for 360 cycles periosteal is applied. In Figure 2C, we show that substantial periosteal bone formation and microdamage in the cortex are generated, when fatigue loading with a peak force of 20 N at 2 Hz until 15% stiffness loss is applied.

### Proteomics changes between normal loading and fatigue loading conditions

The Proteomics software mentioned in the method section reported a comprehensive list of 1,058 proteins that are differentially expressed among normal loading, fatigue loading, and controls. This list was derived from 5,361 IPI-identified Rat proteins observed in the LC-MS/MS experiment of all Rat samples. Among the 5,361 IPI-identified proteins, 578 have Xcorr ='H' (i.e., "high confidence") and 4,783 have Xcorr="L" (i.e., "low confidence"). The 1, 058 differentially expressed Rat proteins can be mapped to 1,171 human proteins using homologous gene mapping methods (see Experimental Procedures for details). Note that only a fraction of these 1,058 proteins may have undergone through real quantitative changes, due to inherent variations of the proteomics platform and the high-variability nature of biological samples.

**Figure 3:**
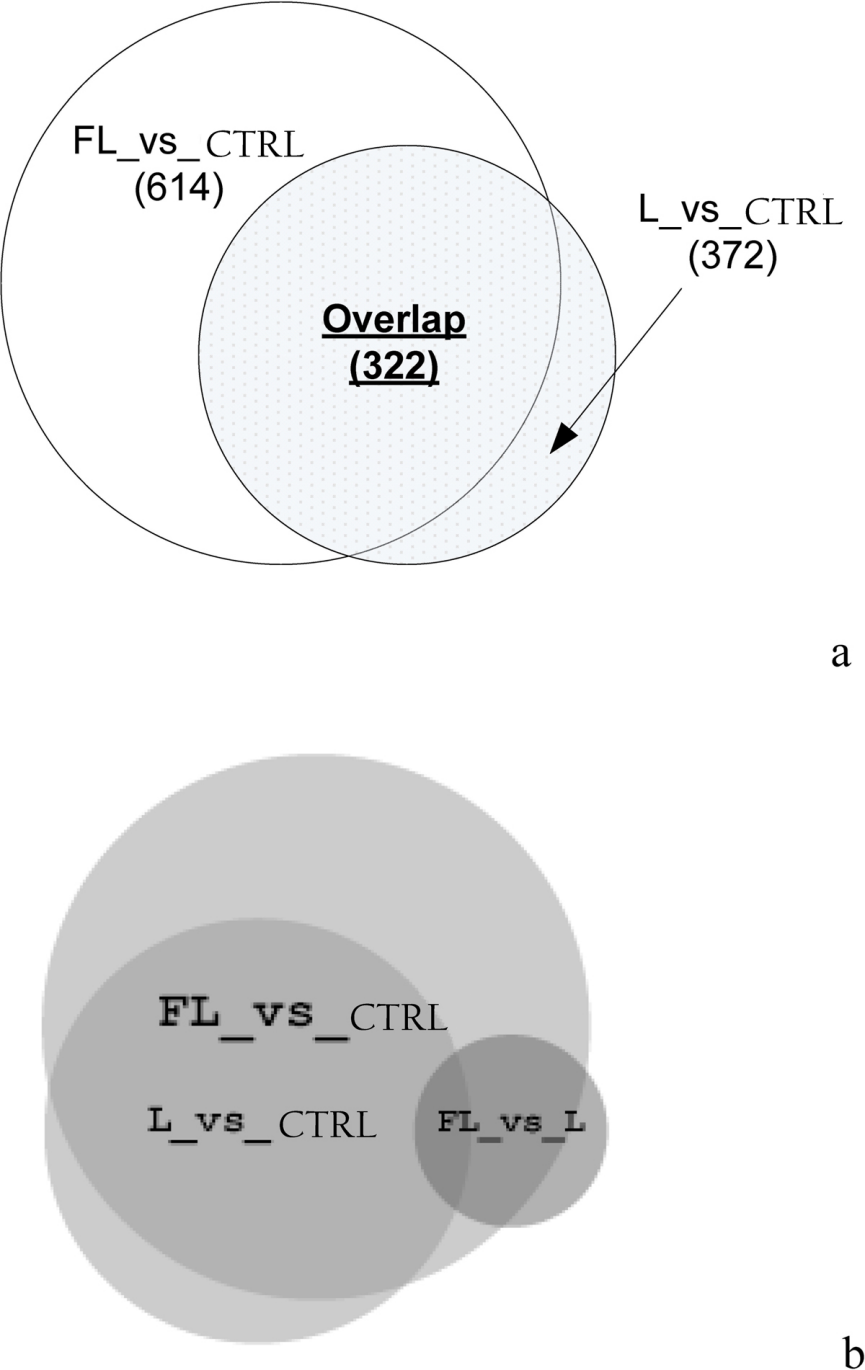
**Venn diagrams showing overlaps between different proteomics comparison results**. a: An overlap of significantly differentially expressed proteins among FL_vs_CTRL, L_vs_CTRL, and FL_vs_L conditions, using filter *F1 *only. b: Overlaps of differentially-expressed proteins among the same set of three types of conditions, using existing filter *F1 *for FL_vs_CTRL and L_vs_CTRL conditions, and a new filter F2 for the FL_vs_L condition. The FL_vs_L total protein set contains 76 proteins, in which only 11 proteins are non-overlapping with the union of proteins in either FL_vs_CTRL or L_vs_CTRL.

In Figure 3, we used Venn Diagrams to show overlaps among three proteomics comparative analysis results, i.e., FL_vs_CTRL (Fatigue Loading against Control), L_vs_CTRL (Normal Loading against Control), and FL_vs_L (Fatigue Loading against Normal Loading), by applying two different types of candidate protein selection filters, F1 and F2 (see Experimental Procedures for details), for results derived from LC-MS/MS proteomics analysis of Rat samples In Figure 3A, only F1 default filter was applied. It showed that there are 322 proteins overlapping between FL_vs_CTRL and L_vs_CTRL proteomics results. Combined together, the two data sets represented 614 + 372 - 322 = 664 total proteins that are quantitatively changed from either loading condition to controls. Note that FL_vs_L produced no "significant" protein list using the standard filter criteria, *F1 *(see Experimental Procedures for details). A plausible explanation is that FL and L are biologically "equivalent" conditions, which make their proteomics level expression indistinguishable. This is very unlikely, since the FL_vs_CTRL and L_vs_CTRL results overlap in significant portions but differently (for FL_vs_CTRL, overalp is 322/614 = 52%, for L_vs_CTRL, overlap is 322/372 = 87%). A second and alternative explanation is that the filter *F1 *may be too stringent (requiring 1.5 fold change differences between loading conditions and controls) to allow detection of quantitative protein expression level changes, which may be quite subtle for FL_vs_L comparisons. Therefore, we applied the second filter, *F2 *(also see Experimental Procedures for an explanation), which provides relaxed (requiring FC≥1.1) yet still statistically significant candidate protein selecting threshold for FL_vs_L differentially expressed proteins. By substituting filter *F2 *for *F1 *in the FL_vs_L condition, we show the new overlapping relationship among FL_vs_CTRL (using the original filter *F1*), L_vs_CTRL (using the original filter *F1*), and the new FL_vs_L (using the new filter *F2*) in Figure 3B. The new Venn Diagram has an added FL_vs_L protein set of 76 candidate proteins. Interestingly, 65 out of the 76 protein (65/76 = 86%) are overlapped with the existing 664 proteins differentially expressed and detected using the stringent filter *F1*. The high degree of overlap resulted in only a slight increase in the final combined data set of 679 candidate rat proteins associated with loading conditions. This observation is consistent with the assumption that applying the F2 filter to the FL_vs_L condition can still control false positives well. However, since filter *F2 *uses a fold change threshold of 1.1--much smaller than the 1.5 threshold used in filter *F1*, we believe that only a subset of the 76 candidate proteins that changed at the subtle amount may have true biological significance.

### Statistical validation of candidate proteins based on correlated loading conditions

To examine how well the quantitative changes measured between FL_vs_CTRL and L_vs_CTRL conditions--a sign that should indicate how consistent and accurate fold changes reported in the proteomics results are, we performed a liner regression on two variables, FC_CTRL_FL as × variable and FC_CTRL_L as y response variable. All the 679 proteins were used but only the data points with both fold change reported were reported. In Table 2, we show the linear regression results, which has an R^2 ^= 0.98. This surprisingly high degree of correlation is perhaps attributable to the commercial operations (use of standard protocols and well-tested proteomics analysis platform that also supports high-volume commercial operations at Monarch Life Sciences). It also supports the use of filter F2 that sets FC threshold at 1.1--a level normally too low to be trustworthy when CV (covariance) of proteomics results are at approximately 15% yet still acceptable for this particular experimental setup, due to high degree of correlations found for fold changes between FL_vs_CTRL and L_vs_CTRL condition.

**Table 2:** Linear regression results of FC_CTRL_FL and FC_CTRL_L variables on differentially expressed proteins in all 3 conditions of the study

*Regression parameter*	*Slope (a)*	*Intercept (b)*	*Data point count*	*R^2^*
**value**	1.09	0.03	679	0.98

**Figure 4:**
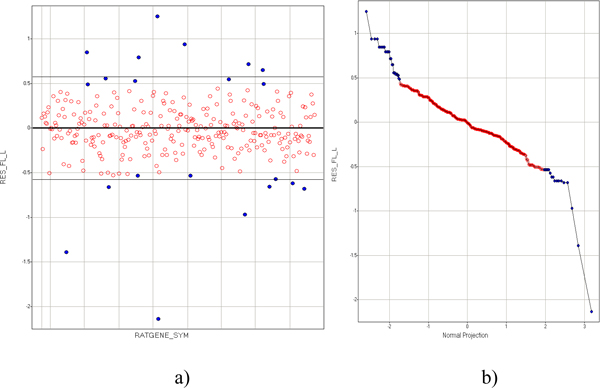
**Determination of outliers in correlated variables FC_CTRL_FL and FC_CTRL_L**. a) Plot of residuals RES_FL_L distributed over each protein identified by Ratgene_sym. The thick line and the two thin straight lines above and below are average and +/-2 standard deviation lines. Residual fold changes for each protein *i *were calculated using the linear regression model shown in Table 2 and calculated using the following formula:*Residue (i) *= FC_CTRL_FL(i) - (a* *FC_CTRL_L(i) *+ *b*), where FC_CTRL_FL(i) and FC_CTRL_L(i) refer to FC for FL_vs_CTRL and FC for L_vs_CTRL for a given protein i, respectively. b) Normal probability plot of residual variable RES_FL_L over normal projection. The outliers are indicated as blue solid dots in both panels. The normally distributed data points are indicated as red empty circles in both panels.

We further analyzed the residual plot for the above linear regression model and determined the normalcy data range (Figure 4). In Figure 4A, we observed that most residuals are evenly distributed within the +/-2.0 standard deviation range (between thin lines), with the exception of several residual extreme values that seemed not normally distributed around the mean (shown as a thick line in the center). To test if the residuals are normally distributed around the mean, we studied the residual normal probability plot (shown in Figure 4B). In regions showing normality, the plot follows a diagonal line. This suggests that residual values in the range vary as expected due to random errors predicted by the linear regression model. Otherwise, we could suspect that the residuals differ from one another by following a different model. In Figure 4B, we observed that the normal probability plot of *Res_FL_L *(Residuals of the FL_vs_CTRL against L_vs_CTRL after fitting the model described earlier) has good normality (linear) in the range of normal projection between -1.85 and +1.85 standard deviations of the mean. Outside this range, the Res_FL_L has a different slope, suggesting non-normality for the outliers from the bulk of data.

### Validated proteomics results -- proteins that quantitatively changed in fatigue loading conditions

Based on the residual distribution and normality probability test results, we reset the data outlier threshold to be within +/-1.85 standard deviation range in the residual plot, with which we narrow down to 42 proteins. Interestingly, the collection of these 42 proteins is a subset of the 76 candidate proteins from the FL_vs_L condition that passed filter *F2*. These 42 proteins correspond to 21 genes, which we showed in Table 3.

**Table 3:** A list of 21 Rat genes whose proteins are found to be differentially expressed with statistical significance between FL_vs_CTRL and L_vs_CTRL conditions

Rat Gene	Human Gene	FC (CTRL_L)	FC (CTRL_FL)	FC (FL_L)	Max Confidence	Peptide Evidence
Capon	NOS1AP	6.72884	6.00145	1.1212	0.98	≥6
Ddx18	DDX18	1.14716	2.13095	-1.85759	0.98	≥6
Ddx21a	DDX21	3.28614	4.10949	-1.25055	0.96	≥6
Fbf1_predicted	FBF1	-3.10292	-2.81444	-1.1025	0.98	≥6
Fcho2_predicted	FCHO2	-1.97277	-2.79227	1.41541	0.98	≥6
Klk14_predicted	KLK14	1.2212	1.88874	-1.54662	0.98	≥6
LOC301506	FSD1	-2.77612	-3.54757	1.27789	0.99	≥6
LOC306805	ASPN	1.83348	2.8254	-1.54101	0.99	≥12
Mrpl45_predicted	MRPL45	2.47117	3.98149	-1.61118	0.99	≥6
Mrpl53_predicted	MRPL53	3.70412	1.94325	1.90615	0.96	≥6
Pdcd8	PDCD8	2.91378	4.15437	-1.42577	0.96	≥6
Pik4cb	PIK4CB	-2.77612	-3.54757	1.27789	0.99	≥6
RGD1562139_predicted	RPL29	2.47771	3.28214	-1.32467	0.98	≥6
Rab40b_predicted	RAB40B	5.42109	4.99103	1.08617	0.98	≥6
Raf1	RAF1	-2.1328	-1.59117	-1.3404	0.97	≥6
Sema5b_predicted	SEMA5B	1.75998	2.60246	-1.47869	0.99	≥6
Serpinb13_predicted	SERPINB13	3.01946	3.82539	-1.26691	0.97	≥6
Slc1a3	SLC1A3	-1.97126	-2.78988	1.41528	0.98	≥6
Slc4a3	SLC4A3	2.15184	1.80834	1.18995	0.96	≥6
Tex101	TEX101	2.007	1.60395	1.25128	0.97	≥6
Upf2_predicted	UPF2	-1.72341	-2.54157	1.47474	0.98	≥6

* "Max Confidence" was calculated as 1- smallest q-value among all the comparison conditions (FL_L, CTRL_FL, and CTRL_L). "Peptide evidence" refers to total number of peptides per group used to calculate Fold Change (FC) and q-value in groupwise comparisons for protein quantifications.

In this table, we can further make several observations. First, protein ranks (indicator of confidence of detection during search) derived from MS search software result as a default is not a reliable predictor for the proteins' biological significance. All significantly differentially expressed proteins in Table 3 have quite low protein ranks, varying between 1500 and 2100. Second, the patterns for differential expression changes are varied from one gene to another. For example, Capon, Ddx21a, Rab40b (predicted), pdcd8, Serbinb13 (predicted) are all induced multiple folds from the resting stage; Fbf1 (predicted), Pik4cb (predicted), Fcho2 (predicted), Slc1a3 (predicted) are all suppressed significantly from the resting stage; and Ddx18, Mrpl53 (predicted), and Mrpl45 (predicted) are all significantly changed for FL_vs_CTRL conditions from L_vs_CTRL conditions. Third, we have shown that at least in some cases, a protein may be significantly differentially expressed in the FL_vs_L condition for many reasons, not necessarily due to a high FC_FL_L, e.g., Capon and Rab40 (predicted)--both due to high FC_CTRL_L and FC_CTRL_FL. Additional details of the protein quantification results for the proteins corresponding to the 21 genes are shown in Supplementary Table 1.

### Activated protein signaling sub-network of molecular response to fatigue loading

We mapped all significant Rat proteins to human proteins using gene homolog matching method describe in the Experimental Procedures. 1,058 significantly changed Rat IPI-identified proteins (using the *F2 *filter on all comparative studies) out of 5,361 IPI-identified Rat proteins from the LC-MS/MS experiment were involved in the mapping. These IPI-identified Rat proteins can be mapped to 513 unique known Rat gene names (the decrease was primarily due to aggregation of proteins isoforms mapped to the same gene). 482 out of the original 513 Rat genes were successfully mapped to 484 human genes using the NCBI Homologene database. The 484 human genes were mapped to 1,171 human proteins identified with UniProt IDs. The slight increase in total protein count from initial 1,058 Rat proteins to 1,171 human proteins suggest that there were a small percentage of one-to-many homologous mapping relationships between Rat and human proteins.

**Figure 5:**
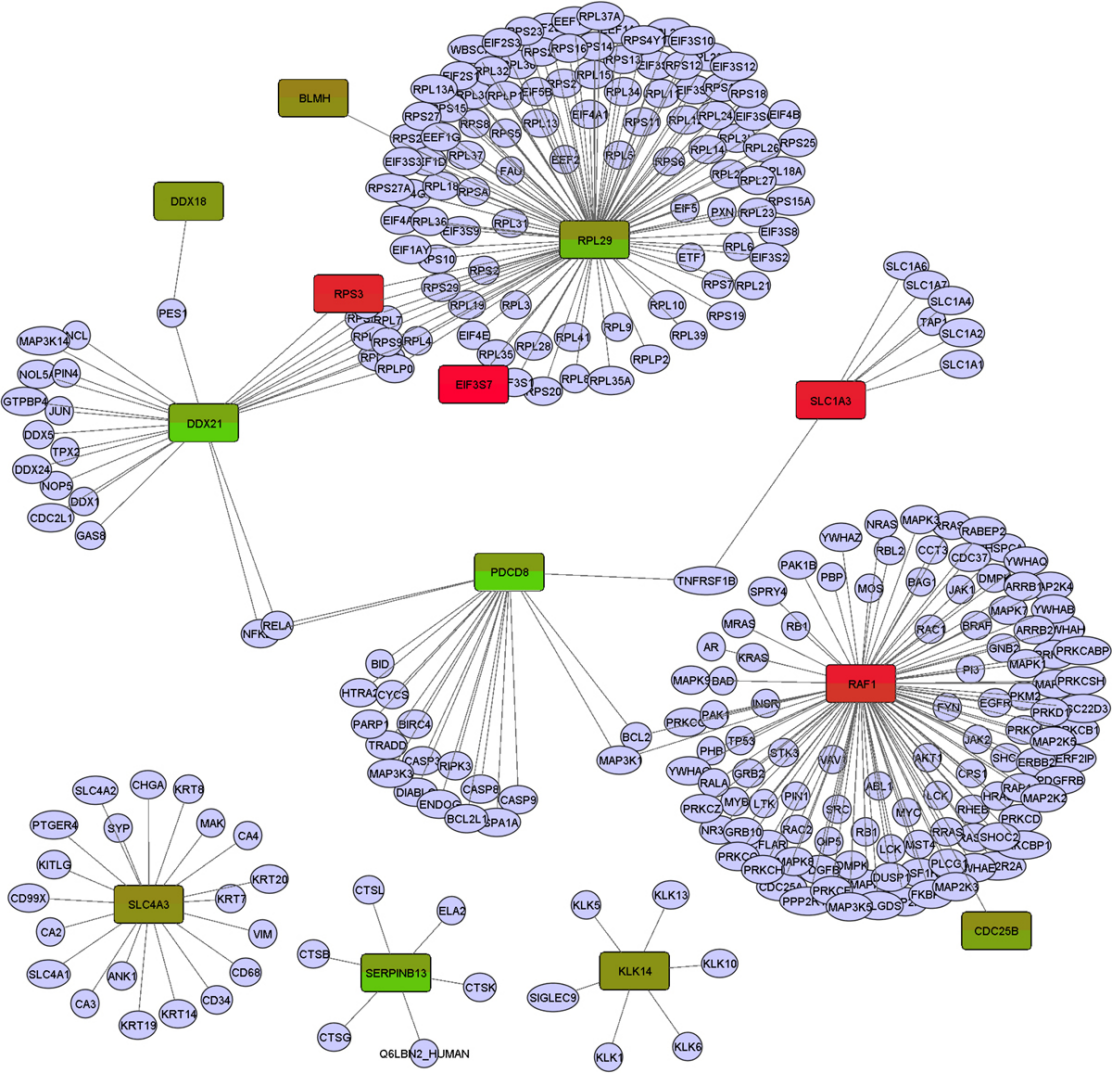
**A protein interaction sub-network of FL_vs_L expanded differentially expressed proteins**. Nodes colored in red or green are FL_vs_L differentially expressed proteins (*seeds*) and nodes in light purple are non-seed expanded proteins recruited through human protein interactions. Edges represent protein interactions recorded in the HAPPI database. Only HAPPI database protein interactions with quality ratings at or above 3 are used. Proteins that are significantly differentially expressed in FL_vs_CTRL or L_vs_CTRL conditions are also shown using the same color legend for FL_vs_L seed proteins, with the rectangle split into two half panels: the upper panel shows the gradient red (FC_CTRL_L >0) or green (FC_CTRL_L <0) colors for the FC_CTRL_L value, while the lower panel shows the gradient red or green color using the same color profile for the FC_CTRL_FL value. Standalone networks with only one pair of interactions are not shown.

Then, using the 42 Rat proteins representing 21 Rat genes (as shown in Table 3) as seed proteins, we built a protein interaction subnetwork. This network represented a coarse biological model that integrated prior knowledge of the functional interaction relationships among proteins and the latest acquired proteomics knowledge on proteins quantitatively changed under fatigue loading conditions compared with normal loading conditions. After the protein interaction network expansion, the initial 42 seed proteins became expanded into a set of 394 human protein interacting pairs covered by 297 human proteins. In Figure 5, we show a visualization of the FL_vs_L expanded human protein interaction sub-network (network with only one pair of interactions are not shown). The largest connected component of this network consists of 9 genes (to be discussed in the next section), which can be used to reason about molecular mechanisms why these proteins changed during mechanical stress conditions that ultimately lead to microdamage in bones.

### Pathway-protein association analysis

The 42 Rat proteins representing 21 Rat genes (as shown in Table 3) were also used to perform pathway-protein association analysis using the Kyoto Encyclopedia of Genes and Genomes (http://www.genome.ad.jp/kegg/) [33]. Significance level for pathway comparisons was set by represented number >3 due to results of small counts. This allows avoiding any assumptions about the shape of sampling distribution of population.

**Figure 6:**
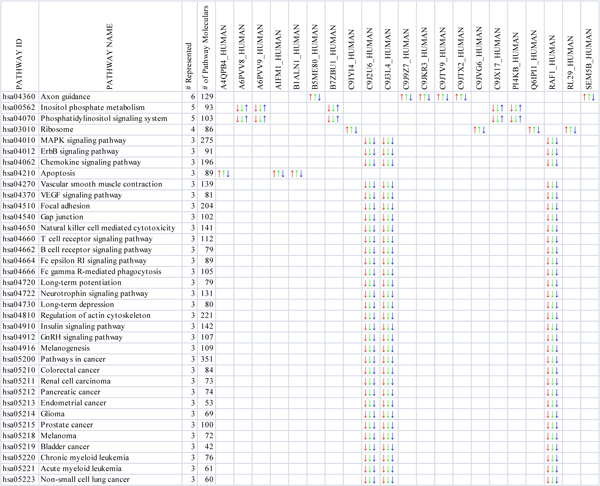
**A pathway-protein association matrix of differentially expressed proteins**. The proteins in the first row from the fifth column to 25^th ^column are differentially expressed with statistical significance between FL_vs_CTRL and L_vs_CTRL. The first column is KEGG pathway ID, the second column is KEGG pathway name, the third column is number of represented proteins in a pathway, and the forth column is the total number of molecular in a pathway. The up-arrow represents up-regulated expression, and the down-arrow represents down-regulated expression. Three comparisons are shown (red for CTRL_L, green for CTRL_FL, and blue for FL_L).

This pathway protein association matrix maps all the biological pathways with pathway proteins. It enriches the top frequent pathways in a given list of pathways, which helps in discovering pathway markers. In Figure 6, 36 pathways and 21 proteins are associated with each other for three comparisons (red for CTRL_L; green for CTRL_FL; and blue for FL_L).

## Discussions

Mechanical stimulation may cause bone cells to express mechano-sensitive genes and proteins through membrane receptors and ion channels and downstream intracellualer signaling cascades [34-36]. These would lead to differentiation of osteoblast progenitor cells and osteoblast prolifeRation [5]. Besides increase in bone formation, fatigue loading produce microdamage [9] in the cortex which also leads to osteocyte apoptosis and further activate bone remodeling through which the damaged cortical bone is repaired [6,37].

In our study, we have found the enhanced expression of proteins involved in receptor binding, RNA processing, cell division and etc. Cell division cycle 25 homolog B (CDC25B), DEAD (Asp-Glu-Ala-Asp) box polypeptide 21 (DDX21), ribosomal protein L29 (RPL29) (seed proteins) and the expanded proteins as shown in Figure 5 were up-regulated. CDC25B that plays a role in cell division seems to allow cell to go into cell division during fatigue loading [38]. DDX21 and RPL29 all are elevated in exercise conditions, and further elevated in fatigue exercise conditions. DDX21 is putative RNA helicase involved in RNA secondary structure alteRation, and Ribosome reassembly [39]. RPL29 is ribosomal protein L29 involved in cell surface hairpin protein binding [40].

NOS (Nitric Oxide Synthase) is increased under the loading condition and further elevated by fatigue loading in this study. NOS is the enzyme to produce Nitric Oxide (NO) in cells [41]. NO has been shown to increase in response to mechanical stimulation in osteoblastic cells [42]. It is also involved in mechanically induced bone formation in vivo [43]. Our study further verifies that NOS may mediate load induced bone formation at the periosteal surface in loading and fatigue loading groups. In addition, the further elevated NOS level under fatigue loading condition suggests NO may also play a key role in mediating the repair of bone damage, such as recruitment of osteoclast precursor, because its actions include changes of the vascular permeability of the damaged area and stimulation of angiogenic activity [41].

Several apoptosis related proteins have been found to change significantly in the current study. Raf1 human (RAF proto-oncogene serine/threonine-protein kinase) was down regulated in the present study. It has a role in the transduction of mitogenic signals from the cell membrane to the nucleus [44]. Raf1 may promot cell survival by antagonizing apoptosis signals-regulating kinase [45]. Our study indicates that loss of Raf1 coincide with increased number of apoptotic osteocytes resulting from fatigue loading, suggesting that Raf1 has a role in protection of osteocytes apoptosis. On the other hand, PDCD8 (Programmed cell death 8) is up-regulated under fatigue loading condition. Because PDCD8 is an apoptosis-inducing factor [46], it may induce osteocytes apoptosis following fatigue loading. Taken together, our study shows that the combination of decreased anti-apoptotic factor, Raf1, and increased pro-apoptotic factor, PDCD8, results in significant increase in the number of apoptotic osteocytes following fatigue loading. Several downstream proteins of Raf1 and PDCD8 pathways, such as Bcl2 and caspase proteins have previously been shown to be involved in osteocyte apoptosis induced by fatigue loading [37,47]. Therefore, this study suggests that drugs targeting on Raf1 and PDCD8 may regulate bone metabolism via prevention of osteocyte apoptosis.

In the pathway-protein association analysis, a list of 42 rat proteins differentially expressed with statistical significance between FL_vs_CTRL and L_vs_CTRL is used to identify topmost frequent pathways. Of the 36 pathways in Figure 6, 13 are related to cancers; 18 to cellular processes (6 immune system, 3 nervous system, 3 endocrine system,2 cell communication, 1 cell growth and death, 1 cell motility, 1 circulatory system, 1 development); 4 to signal transduction; and 1 to carbohydrate metabolism. The top eight pathway are Axon Guidance, Inositol Phosphate Metabolism, Phosphatidylinositol Signaling System, Ribosome, MAPK Signaling Pathway, Erbb Signaling Pathway, Chemokine Signaling Pathway, and Apoptosis. Some of those pathways have been reported to be related to bone metabolism. For example, neural regulation of bone metabolism mediated in osteoblastic and osteoclastic cells via Axon Guidance pathway has been demonstrated in histochemical and pharmacological studies [48] and Togari etc., in their paper, suggested the extension of axons of peripheral sensory and sympathetic neurons to osteoblastic and osteoclastic cells and the possible neural regulation of bone metabolism in these osteogenic cells. Inositol phosphate metabolism and signal transduction pathways was reported to regulate cytoplasmic Ca2+ concentrations in osteoblastic bone cells[49]. In addition, Kennea etc. suggested that there would be robust and functional intrinsic and extrinsic apoptotic pathways in human fetal mesenchymal stem cells or or bone marrow-derived stromal cells which could participate in the repair of mesodermal tissues, such as bone in osteogenesis imperfecta and heart muscle in cardiac ischaemia [50].

Of the 21 proteins, PDCD8 (A4QPB4_HUMAN; AIFM1_HUMAN; B1ALN1_HUMAN) which is up regulated with statistical significance between FL_vs_CTRL and L_vs_CTRL are involved in Apoptosis pathway, RAF1 (C9J2U6_HUMAN; C9J3L4_HUMAN; RAF1_HUMAN) which is down regulated with statistical significance between FL_vs_CTRL and L_vs_CTRL is involved in Cancers, Cellular Processes, and Signal Transduction Pathways. This further indicates the effect of decreased anti-apoptotic factor, Raf1, and increased pro-apoptotic factor, PDCD8, on the increase in the number of apoptotic osteocytes following fatigue loading.

We also found other pathway-protein associations such as PI4KB in Inositol phosphate metabolism and Phosphatidylinositol signaling system pathways, SEMA5B in Axon guidance, and RPL29 in Ribosome. Some of them are linked to bone metabolism or bone formation by previous reports. For example, Miller etc. reported that the presence of HIP/RPL29 during early chondrogenesis is essential for normal skeletal growth and patterning. They designed a ribozyme-mediated knock-down approach to partially down-regulate HIP/RPL29 expression in the multipotent mouse embryonic skin fibroblast cell line C3H/10T To investigate the role of HIP/RPL29 normal expression during cartilage formation [51]. And Mary showed that SEMA5B is a nerve guidance factor which is involved in invasive growth, vascular patterning, axon guidance, and bone development [52].

In addition, Rab40b is a member of Ras oncogene family [53]. Ras oncogenes are small GTP-binding proteins [53]. Besides their role in cell prolifeRation, Ras paradoxically induce both pro- and anti-apoptotic signaling [54]. It remains to be investigated whether Ras plays any role in osteocyte apoptosis following fatigue loading.

There is a possibility that other proteins, such as MRPL45, SLC1A3, UPF2 and ASPN identified in this study are involved in bone response to mechanical loading. ASPN has been found to be related to osteoarthritis [32]. It is remained to be investigated if MRPL45, SLC1A3 and UPF2 as intracellular transports could be stimulated by mechanical stimulation.

In conclusion, using an integrated LC-MS/MS proteomics analysis for the first time in bone mechanical stimulation studies, we have identified several essential proteins related to cell division, which can be linked to osteoblast differentiation and proliferation and bone formation eventually in response to loading. More importantly, our study identified several new proteins associated with osteocyte apoptosis induced by fatigue loading. Our results suggest new insights for future investigation of these proteins as candidate drug targets to regulate bone metabolism and repair bone damage.

## Competing interests

The authors declare that they have no competing interests.

## Authors' contributions

JYC conceived the initial work, designed the method for the data construction. JL implemented the design of bone loading experiments and generated the proteomics data using rat models. FZ collected and analyzed the MS data, performed the statistical analyses. All authors are involved in the drafting and revisions of the manuscript.

## Supplementary Material

Additional file 1 **Protein quantification data for the 21 Rat genes whose proteins levels are significantly changed in Loaded (L) or Fully Loaded (FL) conditions compared with controls (CON)**. "CON_L" refers to comparing L to CON. "CON_FL" refer to comparing FL to CON. "FL_L" refers to comparing L to FL. q-value refers to adjusted p-values. While p-value is an estimate of false positive rate, q-value is an estimate of false discovery rate (FDR). FC refers to Fold Change. "Mean CON/L/FL" refers to mean protein intensities. "%CV Injection" refers to % Coefficient of Variation for injection variation, "%CV Inj + Sample %" refers to the Coefficient of Variation for injection plus sample variation. "# of peptides/group" refers to the number of distinct identified peptides for this protein in any of the three groups: CON, L, or FL. "Mean Xcorr" refers to the mean Xcorr of the peptides identified for this protein. Click here for file
