# Supplementary material for: An integrated proteomics analysis of bone tissues in response to mechanical stimulation
Source: BMC Syst Biol. 2011 Dec 23;5(Suppl 3):S7. doi: 10.1186/1752-0509-5-S3-S7 (PMC3287575; doi:10.1186/1752-0509-5-S3-S7)
Supplement: Additional file 1 — Protein quantification data for the 21 Rat genes whose proteins levels are significantly changed in Loaded (L) or Fully Loaded (FL) conditions compared with controls (CON). "CON_L" refers to comparing L to CON. "CON_FL" refer to comparing FL to CON. "FL_L" refers to comparing L to FL. q-value refers to adjusted p-values. While p-value is an estimate of false positive rate, q-value is an estimate of false discovery rate (FDR). FC refers to Fold Change. "Mean CON/L/FL" refers to mean protein intensities. "%CV Injection" refers to % Coefficient of Variation for injection variation, "%CV Inj + Sample %" refers to the Coefficient of Variation for injection plus sample variation. "# of peptides/group" refers to the number of distinct identified peptides for this protein in any of the three groups: CON, L, or FL. "Mean Xcorr" refers to the mean Xcorr of the peptides identified for this protein. [file 1752-0509-5-S3-S7-S1.doc]

**Additional file 1.** Protein quantification data for the 21 Rat genes whose proteins levels are significantly changed in Loaded (L) or Fully Loaded (FL) conditions compared with controls (CTRL).

“CTRL _L” refers to comparing L to CTRL. “CTRL _FL” refer to comparing FL to CTRL. “FL_L” refers to comparing L to FL. q-value refers to adjusted p-values. While p-value is an estimate of false positive rate, q-value is an estimate of false discovery rate (FDR). FC refers to Fold Change. “Mean CTRL /L/FL” refers to mean protein intensities. “%CV Injection” refers to % Coefficient of Variation for injection variation, “%CV Inj + Sample %” refers to the Coefficient of Variation for injection plus sample variation. “# of peptides/group” refers to the number of distinct identified peptides for this protein in any of the three groups: CTRL, L, or FL. “Mean Xcorr” refers to the mean Xcorr of the peptides identified for this protein.

| **Gene** | **Annotation** | **CTRL_L** | | | **CTRL_FL** | | | **FL_L** | | | **mean CTRL** | **mean L** | **mean FL** | **%CV Injection** | **%CV Inj + Sample** | **# of unique peptides** |
| --- | --- | --- | --- | --- | --- | --- | --- | --- | --- | --- | --- | --- | --- | --- | --- | --- |
|  |  | **q-value** | **p-value** | **FC** | **q-value** | **p-value** | **FC** | **q-value** | **p-value** | **FC** |  |  |  |  |  |  |
| Capon | gi|IPI00325322.3|rs|NP_620277| mass|55864|Rat | 2.90E-02 | 8.14E-04 | 6.73 | 1.96E-02 | 1.13E-03 | 6.00 | 9.79E-01 | 7.23E-01 | 1.12 | 1.21E+05 | 8.12E+05 | 7.24E+05 | 17.79 | 31.51 | 1 |
| Ddx18 | gi|IPI00367078.1|rs|XP_218475|mass|75548|Rat | 4.18E-01 | 2.46E-01 | 1.15 | 1.63E-02 | 3.95E-04 | 2.13 | 9.34E-01 | 1.15E-03 | -1.86 | 2.35E+04 | 2.70E+04 | 5.01E+04 | 12.96 | 20.73 | 1 |
| Ddx21a | gi|IPI00198801.1|rs|XP_228810||mass|85982|Rat | 6.80E-02 | 1.12E-02 | 3.29 | 3.70E-02 | 5.14E-03 | 4.11 | 9.78E-01 | 5.22E-01 | -1.25 | 3.56E+04 | 1.17E+05 | 1.46E+05 | 20.86 | 41.09 | 1 |
| Fbf1_predicted | gi|IPI00370358.1|rs|XP_213526||mass|124310|Rat | 2.59E-02 | 3.50E-04 | -3.10 | 1.71E-02 | 5.69E-04 | -2.81 | 9.78E-01 | 5.55E-01 | -1.10 | 6.31E+04 | 2.03E+04 | 2.24E+04 | 25.29 | 25.29 | 1 |
| Fcho2_predicted | gi|IPI00360301.1|rs|XP_219503|mass|89011|Rat | 4.62E-02 | 4.78E-03 | -1.97 | 1.71E-02 | 5.88E-04 | -2.79 | 9.78E-01 | 6.74E-02 | 1.42 | 2.87E+05 | 1.45E+05 | 1.03E+05 | 5.82 | 24.01 | 1 |
| Klk14_predicted | gi|IPI00367503.1|rs|XP_218641|mass|32271|Rat | 2.72E-01 | 1.25E-01 | 1.22 | 2.05E-02 | 1.28E-03 | 1.89 | 9.34E-01 | 8.04E-03 | -1.55 | 3.33E+05 | 4.06E+05 | 6.28E+05 | 7.10 | 15.36 | 1 |
| LOC301506 | gi|IPI00372515.1|rs|XP_238588|mass|56679|Rat | 2.18E-02 | 1.18E-04 | -2.78 | 9.72E-03 | 3.46E-05 | -3.55 | 9.78E-01 | 7.85E-02 | 1.28 | 2.90E+05 | 1.04E+05 | 8.18E+04 | 11.36 | 15.39 | 1 |
| LOC306805 | gi|IPI00365784.1|rs|XP_225198|mass|30195|Rat | 2.90E-02 | 5.03E-04 | 1.83 | 9.72E-03 | 2.45E-05 | 2.83 | 9.34E-01 | 2.89E-03 | -1.54 | 1.86E+05 | 3.41E+05 | 5.26E+05 | 4.95 | 11.84 | 1 |
| Mrpl45_predicted | gi|IPI00370003.1|rs|XP_213446|mass|35440|Rat | 2.90E-02 | 5.86E-04 | 2.47 | 1.05E-02 | 5.58E-05 | 3.98 | 9.34E-01 | 1.32E-02 | -1.61 | 1.51E+05 | 3.74E+05 | 6.03E+05 | 4.67 | 15.40 | 1 |
| Mrpl53_predicted | gi|IPI00198711.1|rs|XP_342713mass|12735|Rat | 3.68E-02 | 2.51E-03 | 3.70 | 1.35E-01 | 4.50E-02 | 1.94 | 9.78E-01 | 4.97E-02 | 1.91 | 1.42E+04 | 5.27E+04 | 2.76E+04 | 42.57 | 43.00 | 1 |
| Pdcd8 | gi|IPI00204118.1|rs|NP_112646|mass|66723|Rat | 9.18E-02 | 2.04E-02 | 2.91 | 3.97E-02 | 5.91E-03 | 4.15 | 9.78E-01 | 3.39E-01 | -1.43 | 3.31E+04 | 9.63E+04 | 1.37E+05 | 37.66 | 45.56 | 1 |
| Pik4cb | gi|IPI00193760.1|rs|NP_112345|mass|91655|Rat | 2.18E-02 | 1.18E-04 | -2.78 | 9.72E-03 | 3.46E-05 | -3.55 | 9.78E-01 | 7.85E-02 | 1.28 | 2.90E+05 | 1.04E+05 | 8.18E+04 | 11.36 | 15.39 | 1 |
| RGD1562139_predicted | gi|IPI00360888.1|rs|XP_235395|mass|17796|Rat | 4.30E-02 | 4.04E-03 | 2.48 | 1.88E-02 | 1.04E-03 | 3.28 | 9.78E-01 | 2.11E-01 | -1.32 | 1.88E+04 | 4.67E+04 | 6.18E+04 | 7.94 | 26.00 | 1 |
| Rab40b_predicted | gi|IPI00361780.1|rs|XP_221213|mass|31263|Rat | 2.90E-02 | 8.06E-04 | 5.42 | 1.89E-02 | 1.05E-03 | 4.99 | 9.79E-01 | 7.72E-01 | 1.09 | 7.94E+04 | 4.30E+05 | 3.96E+05 | 11.58 | 26.15 | 1 |
| Raf1 | gi|IPI00193150.1|rs|NP_036771mass|72928|Rat | 3.41E-02 | 2.05E-03 | -2.13 | 7.68E-02 | 1.92E-02 | -1.59 | 9.78E-01 | 9.20E-02 | -1.34 | 4.34E+04 | 2.04E+04 | 2.73E+04 | 31.29 | 31.29 | 1 |
| Sema5b_predicted | gi|IPI00365296.1|rs|XP_239438|mass|126504|Rat | 3.01E-02 | 1.30E-03 | 1.76 | 1.19E-02 | 7.38E-05 | 2.60 | 9.34E-01 | 7.81E-03 | -1.48 | 1.79E+04 | 3.14E+04 | 4.65E+04 | 11.18 | 16.79 | 1 |
| Serpinb13_predicted | gi|IPI00371698.1|rs|XP_222497|mass|49806|Rat | 6.57E-02 | 1.01E-02 | 3.02 | 3.28E-02 | 4.14E-03 | 3.83 | 9.78E-01 | 4.58E-01 | -1.27 | 3.80E+04 | 1.15E+05 | 1.45E+05 | 21.45 | 39.21 | 1 |
| Slc1a3 | gi|IPI00324377.1|rs|NP_062098||mass|59697|Rat | 4.62E-02 | 4.79E-03 | -1.97 | 1.71E-02 | 5.88E-04 | -2.79 | 9.78E-01 | 6.73E-02 | 1.42 | 2.86E+05 | 1.45E+05 | 1.02E+05 | 5.96 | 24.16 | 1 |
| Slc4a3 | gi|IPI00210126.1|rs|NP_058745|mass|135407|Rat | 4.09E-02 | 3.40E-03 | 2.15 | 5.58E-02 | 1.11E-02 | 1.81 | 9.78E-01 | 3.29E-01 | 1.19 | 2.02E+04 | 4.34E+04 | 3.65E+04 | 6.31 | 22.01 | 1 |
| Tex101 | gi|IPI00204481.1|rs|NP_620606|mass|27004|Rat | 2.87E-02 | 4.50E-04 | 2.01 | 2.98E-02 | 3.34E-03 | 1.60 | 9.78E-01 | 6.75E-02 | 1.25 | 1.13E+05 | 2.27E+05 | 1.82E+05 | 17.33 | 18.98 | 1 |
| Upf2_predicted | gi|IPI00364924.1|rs|XP_341557|mass|72655|Rat | 7.03E-02 | 1.24E-02 | -1.72 | 1.84E-02 | 9.24E-04 | -2.54 | 9.78E-01 | 4.53E-02 | 1.47 | 1.06E+05 | 6.18E+04 | 4.19E+04 | 8.70 | 21.28 | 1 |
